# Health professionals’ views on maternity care for women with physical disabilities: a qualitative study

**DOI:** 10.1186/s12913-019-4380-y

**Published:** 2019-08-06

**Authors:** Martina König-Bachmann, Christoph Zenzmaier, Barbara Schildberger

**Affiliations:** 1 University of Applied Sciences Tyrol, Innrain 98, 6020 Innsbruck, Austria; 2 University of Applied Sciences for Health Professions Upper Austria, Semmelweisstraße 34/D3, 4020 Linz, Austria

**Keywords:** Birth, Disability, Maternity care, Perinatal care, Pregnancy, Midwife, Obstetrician, Neonatologist, Anesthetist

## Abstract

**Background:**

During pregnancy, childbirth and puerperium, women receive care from a range of health professionals, particularly midwives. To assess the current situation of maternity care for women with physical disabilities in Austria, this study investigated the perceptions and experiences of health professionals who have provided care for women with disabilities during pregnancy, childbirth and postpartum.

**Methods:**

The viewpoints of the participating health professionals were evaluated by means of semistructured interviews followed by an inductive qualitative content analysis of the interview transcripts, as proposed by Mayring.

**Results:**

Four main categories emerged from the inductive content analysis: (i) structural conditions and accessibility, (ii) interprofessional teamwork and cooperation, (iii) action competence, and (iv) diversity-sensitive attitudes. According to the participating health professionals, the structural conditions were frequently not suitable for providing targeted group-oriented care services. Additionally, a shortage of time and staff resources also limited the necessary flexibility of treatment measures in the care of mothers with physical disabilities. The importance of interprofessional teamwork for providing adequate care was highlighted. The health professionals regarded interprofessionalism as an instrument of quality assurance and team meetings as an elementary component of high-quality care. On the other hand, the interviewees perceived a lack of action competence that was attributed to a low number of cases and a corresponding lack of experience and routine. Regarding diversity-sensitive attitudes, it became apparent that the topic of mothers with physical disabilities in care posed challenges to health professionals that influenced their natural handling of the interactions.

**Conclusion:**

The awareness of one’s own attitudes towards diversity, in the perinatal context in particular, influences professional security and sovereignty as well as the quality of care of women with disabilities. There is a need for optimization in the support and care of women with physical disabilities during pregnancy, childbirth and puerperium.

**Electronic supplementary material:**

The online version of this article (10.1186/s12913-019-4380-y) contains supplementary material, which is available to authorized users.

## Background

According to the WHO world report on disability, the prevalence rate of disability in the age group of 18–49 years is estimated at 6.4% in higher-income countries [1]. In Austria, 8.1% of women aged 15–44 years live with permanent impairments [2]. Despite this prevalence, women with disabilities still must face discriminatory situations, such as social exclusion or lack of accessibility [3]. In particular, pregnancy and motherhood among women with disabilities are frequently not taken for granted by their environment or society, and doubt is cast upon their parenting ability [4, 5]. Nevertheless, it has been reported that in Western societies, approximately 5–10% of new mothers live with chronically limiting conditions or disabilities [6, 7].

Women with disabilities had a higher risk of inadequate prenatal care, hospital admissions during pregnancy, cesarean deliveries, preterm deliveries and low-birthweight infants [8]. Regarding interaction with health professionals, they have experienced insensitivity, lack of knowledge about disabilities, limited or inadequate information and support, and discriminatory practices [9].

To assess the current situation of maternity care for women with physical disabilities in Austria, we previously surveyed hospital ward managers for existing structural measures and implemented specific service offerings that ensure accessibility in obstetric wards [10]. While this survey revealed that obstetric departments largely conform to the requirements of the different building regulations, additional measures or adaptations of the inventory for women with physical disabilities were not implemented nationwide.

In a subsequent study, we conducted semistructured in-depth interviews with mothers with motor or sensory disabilities to investigate their personal perceptions and experiences regarding care during pregnancy, childbirth and puerperium. Interestingly, the interviewed women rarely addressed the infrastructural shortcomings but rather expressed a deep need for normality and acceptance as wives and mothers. However, the women experienced limited acceptance of their life choices, a lack of equality, discriminatory attitudes, a lack of support, and a lack of confidence in their ability to be parents in their social environment, which were factors that negatively affected their self-efficacy and self-confidence. Women also reported violations of personal boundaries, a sense of being observed and controlled, and communication with health professionals characterized by mutual fear, insecurity and awkwardness [11].

During pregnancy, childbirth and puerperium, women came into contact with a range of health professionals, particularly midwives. Thus, midwives play a critical role in ensuring that the needs of women with disabilities are met and that the care they receive is individualized and woman-centered [12]. Success in this task requires these women to be considered experts regarding their disability and not a vulnerable group with special needs. Working collaboratively with them will allow the midwife to gain invaluable knowledge [12]. Additional emphasis needs to be placed on teaching health care students about disability, since adequate training of health professionals can inhibit the health disparities of people with disabilities [13].

To extend our knowledge of maternity care for women with physical disabilities in Austria based on these previous findings, we investigate in the present study the experiences and perceptions of health professionals who have provided care for women with disabilities during pregnancy, childbirth and postpartum.

Globally, a limited number of studies have focused on the viewpoint of health professionals with respect to maternity care for women with physical disabilities. McKay-Moffat and Cunningham [14] investigated the experiences of women with mobility-limiting disabilities and of midwives from the same maternity units in the UK. Although all interviewed midwives had provided care for women with disabilities, they generally perceived a lack of knowledge and experience in some aspects of care provision. Despite their generally positive attitudes towards mothers with disabilities, the midwives experienced challenges to effective communication.

In their study, Walsh-Gallagher et al. [15] conducted focus group interviews with health professionals from midwifery, social work and public health nursing in Irish hospitals to explore perceptions regarding how maternity services for women with disabilities can be improved. Consistent with the findings from McKay-Moffat and Cunningham, the health professionals acknowledged their lack of knowledge, competence and skills. Moreover, they concluded that failure to consult and collaborate with the women contributed to a failure to provide individualized woman-centered care for women with disabilities.

Using semistructured telephone interviews with obstetrician-gynecologists and certified nurse midwives with experience providing maternity care for women with physical disabilities in the United States, Mitra et al. [16] investigated barriers to providing maternity care to these women. The reported barriers were assigned to four levels: practitioner level (e.g., lack of training/education, unwillingness), clinical practice level (accessibility), system level (e.g., time constraints, reimbursement policies), scientific evidence level (e.g., lack of disability specific clinical data, lack of guidelines). The authors conclude that there is a need for training, education and practice guidelines regarding maternity care for women with physical disabilities.

## Methods

The present study aims to investigate the viewpoints of health professionals regarding current practice and potential improvements in maternity care for women with physical disabilities in Austria. Using a qualitative study design, the subjective experiences and perceptions of the participating health professionals were assessed by means of semistructured interviews. Data analysis was based on an inductive understanding of research as a meaningful, interpretative scientific process.

### Participants

The selection of the participating health professions is based on the general interprofessional collaboration of midwives, obstetricians, neonatologists and anesthetists in the obstetric setting. Recruitment took place through personal contacts. Health professionals were excluded from participation if they felt they had too little experience caring for women with disabilities. In total, semistructured interviews with seven midwives and six medical doctors (two obstetricians, two neonatologists and two anesthetists) were conducted between January 2017 and January 2018.

### Interview procedure

In preparation for the interviews, the research team developed an interview guide. In the course of the semistructured interviews, the viewpoints of the health professionals regarding the provision of care for women with physical disabilities and specific aspects of their maternity care were of particular interest to reveal potential enhancements and improvements of the care provided. Specific questions about women with physical disabilities and their special needs were asked. Furthermore, participants were surveyed regarding how networking with other disciplines can lead to success and possibly be improved. For example, were there any prejudices, uncertainties and/or fears of their own in the care of these women, and what should be avoided in care practices? Can further training, improved equipment or an extended range of services facilitate work?

In the time preceding the study, two pilot interviews with midwives were conducted, and the interview guide was subsequently adapted based on the findings of this pretest. The adapted interview guide is given in Additional file 1. After written consent was obtained from participants, the semistructured interviews were conducted with the health professionals at their respective institutions. Interviewers were not employed at the same institutions as the interviewees, thus ensuring professional distance. The interviews were audiorecorded with the permission of the participants. The duration of the interviews ranged from 15 to 30 min.

### Data analysis

The audiorecorded interviews were transcribed and pseudonymized. Participants were pseudonymized as follows: midwives as M1, M2, …. and M7, obstetricians as O1 and O2, neonatologists as N1 and N2, and anesthetists as A1 and A2. The transcribers signed a confidentiality agreement, and the original audio recordings were deleted after transcription of the interviews.

The analysis of the transcribed interviews was performed by qualitative content analysis according to Mayring [17, 18]. The aim of this rule-governed analysis procedure is to create order-building categories and to filter and interpret the data accordingly. Therefore, the transcripts were analyzed by the stepwise inductive construction of codes, which were subsequently sorted into main categories and subcategories. The goal was to consider all of the remarks with open coding, following a strictly inductive approach. The categorization was performed in several iterative steps, each with immediate reference to the material collected. To enhance the trustworthiness of the results, a second researcher independently performed coding, and occasional differences in the researchers’ conceptions were discussed and resolved within the research team.

## Results

From the qualitative inductive content analysis of transcripts from the 13 interviews conducted, four main categories could be identified: (i) structural conditions and accessibility, (ii) interprofessional teamwork and cooperation, (iii) action competence, and (iv) diversity-sensitive attitudes.

### Structural conditions and accessibility

The care of women with disabilities requires individual care measures and strong flexibility beyond routine procedures. Both intramural and extramural care must be adapted to the needs of these women. Lack of structural services and lack of time resources demand a high degree of improvisation from the professionals to find solutions to assure high-quality care.

The interviewees stated that the structural and organizational condition of obstetrics is poorly suited to adequately care for women outside a routine concept. For example, several participants highlighted a lack of accessibility to doctors’ offices in the extramural care, which limited the free choice of obstetricians or pediatricians. The specific barriers addressed were a lack of construction conditions, such as elevators, wheelchair ramps or wheelchair-accessible restrooms, and a lack of orientation aids for women with sensory impairments.

*M1: ‘As far as registered doctors are concerned, there is no accessibility in any case. The women have to choose the gynecologist according to the construction situation and not according to sympathy.’*

*N1: ‘Well, unfortunately we are not yet properly adjusted for the blind, i.e., with Braille, starting at the doorbell and so on.’*

One midwife also addressed that the women with disabilities sometimes did not appear to be well-prepared for birth due to the lack of availability of accessible antenatal classes. Women with physical disabilities might particularly benefit from domiciliary visits at their accordingly adapted homes.

*M1: ‘What many women need is rather that there is a mobile offer…so that they don’t have to drive elsewhere, but that someone comes into the house, which then doesn’t cost much extra and isn’t connected with a big “just for your sake” or that they have to beg for it, but rather that it is a matter of course.’*

Construction-related barriers were also frequently mentioned in the intramural area. These included infrequent accessibility of restrooms and bathrooms, rooms that were too small and irregularly shaped, doormats on which wheelchairs could get stuck and wheelchair-suitable room furnishings. Moreover, a lack of specific aids, such as height-adjustable changing tables or vibrating/flashing baby monitors for parents with hearing impairments, was addressed.

*M5: ‘We had to cover a longer distance until we had a wheelchair-accessible restroom and, for example, until she had the possibility of taking a shower after birth, yes.’*

*O2: ‘If the spatial adaptation would be a bit better, even in hospitals with outpatient rooms, where you might be able to drive in not only the smallest wheelchair. With the possibilities of repositioning and accessible toilets, not only every few kilometers…I think that would really be a relief.’*

Several interviewees expressed a greater need for privacy and an increased amount of time required when caring for women with physical disabilities. For example, a participating midwife wished for an extra room in the outpatient ward, and a colleague focused on privacy in intramural care:

*M1: ‘I need a one-on-one situation for a consultation, an examination. The normal round, how many people are going? And I don’t need a roommate, for example, when it comes to my urinary bladder, that’s nobody else’s business.’*

The increased time requirements were mainly attributed to understanding specific needs when caring for complex cases. To meet these requirements, participants wished for organizational adaptations, such as timely information, more time to care for women with physical disabilities, more staff when needed and continuity of care.

*A1: ‘I also think that we should, in any case, be informed when these patients arrive, when they become known in the system. We should then have contact with them and be able to discuss what we should do when they are in pain.’*

*N2: ‘The number of staff is of course already quite small, and if a mother needs additional help at the mother-child ward, then that is of course already a certain requirement, where you might need more staff in that specific case.’*

*M2: ‘Structurally? More time for the care and immediate introduction of the pregnant woman in the delivery ward, not only in the outpatient department.’*

In this context, two participants expressed their desire for a single point of contact or a specific platform that provides information on care for women with physical disabilities during pregnancy and childbirth. Restrictions in structural and organizational conditions also might complicate the effectiveness of communication that often depends on special services.

*N1: ‘At the moment, I have only one deaf woman who has already come perfectly provided with an interpreter who has thankfully translated everything for us. The logistical problem that arose for us was how to contact this mother or how the mother can contact us by telephone or at all if there is no interpreter available?’*

Thus, accessibility is not only guaranteed by the structural adaptation of the departments but also requires comprehensive structural and organizational adjustments. In this sense, low-threshold care services are designed in such a way that they are accessible for all target groups. Low thresholds require consideration of temporal (e.g., opening hours, avoidance of waiting times), spatial (accessibility), content (e.g., individually adapted care) and social (e.g., anonymity, voluntariness, noncommittal) dimensions.

*A1: ‘The associated tasks that we have had have involved, on the one hand, the fact that one first had to become aware of the wheelchair-dependent patients and what their medical requirements were.’*

*O2: ‘...but it must be low-level access...not only at certain times.’*

### Interprofessional teamwork and cooperation

In caring for mothers with disabilities during pregnancy, childbirth and postpartum, interprofessional cooperation is of great importance. Successful teamwork supports one’s own security and sovereignty in actions and decisions [19]. To meet this requirement, the interviewees reported the importance of strong networking with other disciplines when working with mothers with physical disabilities. In this context, the availability of experts provided reassurance and security to health professionals when caring for mothers with physical disabilities. Interprofessionalism means a complex process of collaboration between different disciplines and professions with the aim of establishing the best possible care, based on a common knowledge base [20].

The majority of the interviewed health professionals emphasized the teamwork within the obstetric core team, namely, the interaction with obstetricians or midwives. In this respect, some health professionals mentioned the importance of the instant and individually coordinated care of the women with the obstetric team.

*M2: ‘It would be ideal if, in every labor room, there were the possibility of individual care by a team of midwives and gynecologists that...was already in contact before, during pregnancy.’*

*A2: ‘It has always been necessary to somehow—by getting to know each other, discussions with colleagues, especially obstetricians, but also with the patients and their partners—determine the strategy for childbirth.’*

Regarding cooperation with other professions, the participants used differing approaches. Some health professionals did not use large networks in their daily practice but rather, if necessary, involved a social worker to initiate further steps. On the other hand, several interviewees were in contact with various organizations and professions. In particular, midwives who were involved in puerperal care at the respective homes of their clients reported that they sometimes consider bringing in social and psychological support when caring for women with physical disabilities. In this regard, social work, Catholic charities, the National Center for Early Childhood Intervention and psychologists were addressed. Additionally, the interviewed midwives emphasized cooperation with disability associations, such as the Austrian Federation of the Blind and Partially Sighted or the Austrian Federation of the Deaf Austrian, and the need for sign language interpreters. The participating neonatologists also mentioned the involvement of social work and disability associations. Moreover, one neonatologist stressed the importance of speech and language therapists.

*N1: ‘It should also not be forgotten that special support may also be necessary for the children, for the babies. For example, for this child I care for, both parents are deaf and speech-impaired. Nevertheless, it is very important for the child to hear spoken language in order to learn it. Thus, from a very early age, the child will receive early language support.’*

The interviewed anesthetists mainly referred to obstetricians as their contact persons when caring for women with physical disabilities during childbirth. The obstetricians themselves addressed the potential involvement of health professionals from various disciplines, such as midwives, anesthetists, neonatologists, urologists, rheumatologists, neurologists, physical therapists and dietitians, as well as social workers.

*O2: ‘…that you know who you can call, who you can contact and, of course, that the people are willing to provide a specific service then. This can range from dietetic counseling to physiotherapy and social counseling.’*

Also mentioned were networks in the form of interprofessional teamwork, with the aim of exchanging experiences and analyzing, reflecting on and discussing possible improvements. Ideally, this exchange could be implemented in the form of a quality circle or platform.

*M1: ‘Exchanging information with others who are also affected, such that we, as an all-around obstetric group, know what there is.’*

*M3: ‘That would be the first thing that would come to my mind and that one would more often talk about, or something like that, in staff meetings: what special women did we have in the last month, in the last two months, and what was there.’*

*N1: ‘It would be important...to have such a central contact point where you can get tips when you need them...If there were any such committee, it would be great to say that the information is bundled there.’*

### Action competence

The term action competence describes the ability to solve problems independently and professionally in appropriate situations and comprises the dimensions of professional, personal and social competence [21]. It serves as a method of coping with professional tasks and challenges [22].

Reported shortcomings in action competence could primarily be ascribed to two areas: on the one hand, a lack of routine in the care of mothers with disabilities, and on the other hand, a lack of specialist knowledge about the obstetric relevance of specific symptoms related to disabilities. These aspects could lead to fears and insecurities among health professionals. Fear and insecurity can impede optimal care processes and thus result in an increased risk of an undersupply but also a rapid pathologization in the course of care during pregnancy, childbirth and puerperium. The interviewees named insecurities and inhibitions in concrete action and even a feeling of shame that they could not adequately do justice to this situation.

*M4: ‘A primary insecurity is there, because one simply is not often confronted with it, and if one then notices that one does not progress well with what one tries, the insecurity does not become smaller and at the birth itself, an exceptional situation, where often simply no progress at all is possible anymore.’*

*M1: ‘Yes, we can definitely be much more courageous. To be much more courageous, much more direct....There is certainly a lack of confidence to ask questions.’*

*M3: ‘...maybe I could also embarrass myself as a helping person...if you’re in need of help yourself.’*

*M5: ‘Yes, she was actually very clear in her statements, but my uncertainty did not allow me to ask her that directly in advance.’*

In addition, situations and processes were described in which the health professionals became aware of their own deficits in their action competence. In this context, the positioning of women with motor disabilities, the instruction of a blind woman in changing the newborn, and sensitivity training for paraplegic women to perceive contractions were cited as challenges. Moreover, several participants addressed insecurities regarding the use of disability-mediating medications during pregnancy and interactions of anesthesia with disability symptoms.

*M2: ‘There are often uncertainties in drug therapies. What can the woman continue to take during pregnancy, what does she have to take?’*

*A1: ‘What I always notice with these patients is that there is often uncertainty, especially in medical matters. What do you have to do? For example, there is a patient, a caesarean section, a wheelchair user, where we are then called. How do we do there? Is it possible to do an epidural do we have to do a general anesthetic?’*

*M5: ‘Here it is important that I have the knowledge in advance. For example, is pain management different in a woman with paraplegia? What do I have to consider in particular? And how can I counsel a woman during pregnancy?’*

On the other hand, several participants acknowledged their lack of specific knowledge and openly and without embarrassment discussed options with the women, thereby avoiding insecurities and fears.

*M1: ‘So I put my cards on the table and say: I have never cared for a woman in a wheelchair before, you are the first. I am quite grateful to you when you tell me what you can do, and what you need from me, that we know how things stand. So in that sense, I have not felt insecure at all.’*

*M3: ‘Well, I’ve always tried to ask people a lot, what’s the problem now, and I said, look here, usually they do that one way or another, does that work for you as well? So then we’ve often talked about it very openly or the women have talked about it themselves when they’ve noticed that I’m trying to adjust, telling me exactly what their difficulties are.’*

*M5: ‘I would approach the woman quite differently and ask what she needs from me, that she supports me so that it is pleasant for her and today I also know that these women are very open.’*

The ability to research, connect, expand, evaluate and integrate subject-related and interdisciplinary knowledge into larger contexts is an essential aspect of action competence [22]. Knowledge is an elementary precondition for action competence and in turn restricts it if specific knowledge in a subject area is inadequate. The assessment of one’s own level of knowledge is a prerequisite for initiating improvements in action [23]. The following quotes demonstrate the high level of awareness of the health professionals surveyed with regard to their professional competence and lack thereof.

*N2: ‘Yes, as far as knowledge is concerned, one is of course always of a certain, how shall I say, obligation to provide. So I have to read it myself.’*

*A1: ‘What I have always noticed is that when we have such patients, there is often uncertainty, especially in medical matters, such as how do we do there, what we have to do there, because we then discuss this just within the anesthesia department.’*

*O1: ‘To be honest, I had to read myself...I never had anything to do with the symptoms before, and I told her [the woman] that I have to read myself.’*

For optimal care, the active involvement of affected women was mentioned. In this way, women became experts in their disability and could provide, based on their personal experience and knowledge, a significant contribution to the creation of an individual treatment concept.

*O2: ‘So I think it’s impossible to take care of a woman with a disability during pregnancy and not put much more work, energy, readiness to read, etc. into it....I also try to tell the students as doctors in training that if you really want to learn something, then listen very well/exactly to what those affected say, because they have a lifelong experience of illness, they can give you tips....I mean, you can never be so competent in all disciplines....I mean, it is relevant, regardless of one’s own insecurity and fears, to the situation...’*

*M7: ‘…and that wasn’t at all alarming then because I realized there’s just so much more competence there in this family.’*

### Diversity-sensitive attitudes

Heterogeneity often harbors the risk of social categorization and stereotyping, which can lead to prejudice and discrimination. A diversity-sensitive attitude allows for the breakup of this process and the prevention of discrimination through mutual respect and attentiveness.

*O2: ‘I mean, this defensive reaction that women sometimes experience is mainly due to the fact that they simply do more work, yes.’*

In their statements, the health professionals contended with the accuracy of their actions. Their answers illustrated the variety of different attitudes as well as the internalized images of disability that influenced care.

*N2: ‘Prejudices are probably an expression of ignorance and insecurity.’*

*M3: ‘...so there are images that do not correspond to reality...’*

*M6: ‘I just...believe that you just have to be ready somehow to get involved. That’s what I think is the essential thing.’*

*M3: ‘...then nobody wants to go to her [the woman] because she is difficult. That is, she actually gets less attention and devotion, and nobody really wants to get involved, and it is just hard...’*

*O1: ‘No, not prejudices in that sense. I actually admired them for the fact that they dare to do that and how well they actually do it.’*

Particular emphasis was placed on the importance of sensitive communication and interaction, taking into account a positive approach to diversity perspectives. The ability to reflect, mindfulness, empathy and intuition are important aspects to be considered in a diversity-sensitive care context. In the interviews, it was noted, for example, that women’s competence in their maternity care are to be recognized and strengthened.

*O1: ‘No, I think that this is actually the most important thing for these women—to really provide continuous care, and on one level, I think that this is the most important thing and simply take them seriously and make sure that everything is done to give them these missing competences or possibilities that they have—that they are simply given aids so that they can master this a little better.’*

The complexity of the issue is reflected in the challenge of verbal and nonverbal interaction with mothers with disabilities and the possible lack of acceptance of motherhood.

*O2: ‘Yes, no, it’s the thought that sometimes comes: does she have to get pregnant now? So she is also someone who, let’s say, is kept relatively laboriously in a functioning life and then sets this desire to have children so high in the hierarchy of her values, thereby endangering some things and also, one then also thinks of what kind of environment the child should live in.’*

Moreover, the issue of prenatal diagnosis in expectant parents with disabilities was presented as an ethically and morally discussed dilemma by an interviewee.

## Discussion

In the present study, the experiences and perceptions of health professionals who have provided care for women with physical disabilities during pregnancy, childbirth and puerperium were investigated via qualitative inductive content analysis and assigned to four main categories: (i) structural conditions and accessibility, (ii) interprofessional teamwork and cooperation, (iii) action competence, and (iv) diversity-sensitive attitudes.

According to our findings, the structural conditions were frequently not suitable for providing targeted group-oriented care services due to limited accessibility and lack of specific aids. In addition to structural conditions, shortages of time and staff resources also limited the necessary flexibility of treatment measures in the care of mothers with disabilities. These deficits resulted in a lack of attention in care. These findings might indicate healthcare disparities and demonstrate that women with physical disabilities are still likely to be disadvantaged regarding participation in the health system. All services offered should be nondiscriminatory, low-threshold and self-evident.

The states parties to the UN Convention on the Rights of Persons with Disabilities according to Article 23 commit themselves to ‘take effective and appropriate measures to eliminate discrimination against persons with disabilities in all matters relating to marriage, family, parenthood and relationships, on an equal basis with others’ [24]. Austria has ratified this convention, and in 2012, the government adopted the National Action Plan on Disability 2012–2020 as a long-term strategy for its implementation. The National Action Plan contains 250 measures that must be realized by 2020.

In Austria, social insurance is organized based on the compulsory insurance principle. Compulsory health insurance applies to almost all employees, self-employed persons, pensioners, persons claiming unemployment benefits and dependents of these groups. The compulsory health insurance-covered maternity care consists of several antenatal examinations and offers one antenatal consultation by a midwife. Home birth or giving birth at birthing centers is covered for low-risk pregnancies. However, the vast majority of births (> 98%) take place at hospitals. Postnatal care is provided either at hospital wards or at home by a midwife until the fifth day after birth and thereafter, in case of further need according to the midwife. Additional examinations or offers are covered by additional voluntary private health insurance or are at the expense of the women themselves [11].

Our findings demonstrated that the care of women with physical disabilities is frequently accompanied by increases in workload due to preparatory research, potential communication difficulties or specific measures. Thus, an adequate care might require additional resources. However, the rapidly increasing expenditure in the health sector has forced health care providers to restrict costs through business management thinking and entrepreneurial strategies. With regard to the change from health service to health economy, management concepts such as controlling, cost management, factoring, leasing and process optimization are areas of greater focus [25]. Although this focus constitutes a health policy necessity to provide care for the population, it is necessary to ensure that processes and structures can be flexibly adapted to suit the situation and requirements. Individually adapted care outside the care routine contributes not only to quality assurance but also to the satisfaction of those affected.

The second identified main category emphasized the importance of interprofessional cooperation for adequate care highlighted by the interviewees. Interprofessionalism was perceived as an instrument of quality assurance and ensured direct networking with adjacent disciplines, institutions and federations in Austria, e.g., the Austrian Federation of the Blind and Partially Sighted and the Austrian Federation of the Deaf or the National Center for Early Childhood Intervention. Team meetings were addressed as an important component of high-quality care.

A mindful and active interprofessionalism has been shown to increase patient safety. Various studies have demonstrated that critical events in patient care are due to a lack of communication and a loss of information [26, 27]. Mutual narration in a team increases trust, the recognition of different job-specific perspectives, and the subsequent development of a consensus. The retrospective analysis and reflection of events is helpful for discussing improvements in care and developing guidelines. In this respect, the statements of the interviewees demonstrated that they recognize the importance on learning from each other and from the different competences and perspectives of the different professions involved.

How the composition of interprofessional teams is designed depends on the specific requirements of each individual task. Adequate context conditions (e.g., clear tasks, clarification of roles and competencies, training, flow of information, documentation) have a major influence on the success of the team [28].

The third main category action competence implies the dimensions of professional, personal and social competence. An essential component of action competence is problem-solving competence, i.e., the ability to react adequately to deviations from the norm through a systematic approach and to develop solution strategies for the corresponding situation.

In the interviews, a lack of action competence was attributed to a low number of cases and a lack of experience and routine. Particularly in medicine and health care, it is necessary to be well-prepared for rare events to be able to act competently in this situation [29]. The development of guidelines, the organization of training and continuing education can play a vital role in helping to build action competence [30]. In this respect, the interviewed health professionals also expressed their desire for platforms for interprofessional exchange of experiences and for specific points of contact that provide information on care for women with physical disabilities.

Reflection on one’s own action competence enables professionals to recognize their own knowledge, identify deficits and deduce improvements. From our findings, it becomes clear that this process of self-assessment can lead to a feeling of helplessness and excessive demands. The presence of fear, insecurity and lack of knowledge on the part of health professionals and the possibility of embarrassment on their part if they need to ask for help seem to be related to the fear of not knowing information. The underlying fear of making mistakes and the fear of violating the autonomy and self-determination of women with disabilities must be transformed into a trusting and powerful relationship [12]. To make this possible, training and advanced education are needed to strengthen health professionals.

The acknowledgement of one’s own deficits and lack of knowledge towards the women and the inclusion of their specific knowledge regarding their disabilities and needs allowed the participating health professionals to avoid the experience of insecurities and fears. This finding is in accordance with previously reported viewpoints of women with disabilities, who generally appreciate having their health professionals acknowledge that they do not know something rather than to act as if they do know. On the part of the health professionals, women reported a lack of knowledge about their specific needs related to pregnancy and perceived failure to consider their knowledge and expertise related to their own disabilities [31, 32].

The fourth identified main category summarizes diversity sensitive attitudes. Diversity is an expression of social heterogeneity and subsumes the core dimensions: gender, age, origin and ethnicity, disability, sexual orientation, religion and ideology. The concept of diversity management aims to provide equal opportunities for the participation and integration of all members of a community. A diversity-sensitive attitude thus helps to identify unequal and discriminatory conditions and create equal opportunities [33]. As a strategy, diversity management thus aims to reflect on interaction and manners and to sensitize, flexibilize and expand the employees’ capacity for perception, communication and action [34].

In the course of the conducted interviews, it became apparent that the topic of mothers with physical disabilities in care posed challenges to health professionals that influenced their natural handling of the interactions. The recognition of one’s own ideas and prejudices is the prerequisite for action that is open and oriented towards women’s needs. The results also demonstrated that the discourse on the social inclusion of individuals with disabilities and on parenthood with disabilities must be continued. More importance must be given to the willingness to reflect with regard to prejudice and diversity sensitivity to further reduce restraints and fears of contact. The prejudices of health professionals can influence the quality of care, which can result in the oppression, isolation and marginalization of women. In this regard, it was reported that health professionals focus more on the women’s impairments than on the available resources and possibilities. They are treated more with skepticism, surveillance and control. The women should not be considered a vulnerable group but rather as experts in their disabilities [12].

**Fig. 1:**
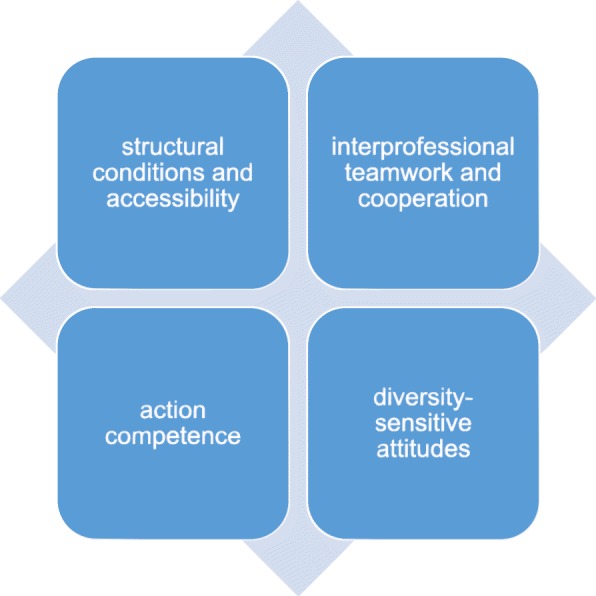
Different dimensions of maternity care for women with physical disabilities from the viewpoint of health professionals

The main categories identified in the present study, namely, structural conditions and accessibility, interprofessional teamwork and cooperation, action competence, and diversity-sensitive attitudes, are closely interlinked, mutually dependent and influence each other (Fig. 1). In this way, individual attitudes towards diversity impact action competence, interprofessional teamwork and the level of structural conditions and vice versa; that is, framework conditions can affect employees’ attitudes and limit and/or expand their action competences.

Our findings are in agreement with those of previous studies that reported health professionals’ acknowledgement of their lack of knowledge, competence and skills [14, 15]. To improve the maternity care provided for women with physical disabilities, there is a need for adequate practice guidelines and for the training and education of health professionals [16]. In this respect, the WHO provided information on better health care for people with disabilities in the World Report on Disability [1] and made the following concrete recommendations: ‘*To improve health service provider attitudes, knowledge, and skills, education for health-care professionals needs to contain relevant disability information. Involving people with disabilities as providers of education and training can improve knowledge and attitudes. The empowerment of people with disabilities to better manage their own health through self-management courses, peer support, and information provision has been effective in improving health outcomes and can reduce health care costs’* [1].

The present study has several limitations. The relevance of the findings is limited by its small sample size. Furthermore, it is possible that the health professionals who agreed to be interviewed who have a higher sensitivity for this topic and thus a higher awareness of the problem, and therefore, they are likely not representative of the occupational group. To increase the credibility and validity of the results, several health professions were included in the study, and two researchers performed the analysis independently (data and investigator triangulation). However, additional procedures might have enhanced the trustworthiness of the findings; for example, participants were not asked to review a summary of the findings. Moreover, the duration of several interviews (with midwives) was rather short. This circumstance affected the interviews with midwives who turned out to have limited practical experiences in caring for women with physical disabilities. The findings of our study are reflective of a particular cultural and temporal context and thus might not be generalizable to other people or other settings, particularly countries with different maternity services.

## Conclusions

Our findings indicate the importance of the compliance with and implementation of adequate and situation-adapted structural conditions and the establishment of accessibility. Interprofessional teamwork and cooperation, action competence and diversity-sensitive attitudes are fundamental prerequisites of adequate professional maternity care for mothers with disabilities. The awareness of one’s own attitudes towards diversity, in the perinatal context in particular, influences professional security and sovereignty as well as the quality of care of women with disabilities. According to these findings, there is a need for optimization in the support and care of women with physical disabilities during pregnancy, childbirth and puerperium.

## Additional file

Additional file 1: Interview Guide. (PDF 99 kb)

## Data Availability

The data analysis is based on individual interviews that, due to ethical approval, are only available to the researchers involved in this study.
