# Supplementary material for: Health professionals’ views on maternity care for women with physical disabilities: a qualitative study
Source: BMC Health Serv Res. 2019 Aug 6;19:551. doi: 10.1186/s12913-019-4380-y (PMC6685240; doi:10.1186/s12913-019-4380-y)
Supplement: Supplementary file 1 — Interview Guide. (PDF 99 kb) [file 12913_2019_4380_MOESM1_ESM.pdf]

Additional File 1: Interview Guide

- You have assisted women with physical disabilities in obstetrics.

  o What type(s) of disability did these women have?

- In which phase have you assisted the women? (Pregnancy, childbirth, puerperium?)

- Which other professions or disciplines (persons) are or can be involved in the care of women with physical disabilities at your facility?

  o e.g., social workers, disability care workers, gynecologists, obstetricians, anesthetists, neonatologists, physiotherapists, occupational therapists, masseurs…

- Do you use specific networks to care for women with physical disabilities?

  o e.g., via disability organizations...

- Do you experience your own insecurities or fears when caring for women with physical disabilities?

- Do you think one's own prejudices could have an impact at the workplace?

- In which context do you experience insecurities or fears with respect to women with physical disabilities?

- What do you think should be avoided when caring for women with physical disabilities?

- What do you need in order to be able to safely (competently, sufficiently, according to their needs,...) care for women with physical disabilities?

  o Structurally?

  o Equipment?

  o Knowledge, competence?

  o Service offering?
